# Catheter-Associated Urinary Tract Infection and Obstinate Biofilm Producers

**DOI:** 10.1155/2018/7624857

**Published:** 2018-08-26

**Authors:** Govinda Maharjan, Priyatam Khadka, Gomik Siddhi Shilpakar, Ganesh Chapagain, Guna Raj Dhungana

**Affiliations:** ^1^Janamaitri Foundation Institute of Health Science (JFIHS), Kathmandu, Nepal; ^2^Department of Microbiology, Tribhuvan University Teaching Hospital, Kathmandu, Nepal; ^3^Department of Pathology, Tribhuvan University Teaching Hospital, Kathmandu, Nepal

## Abstract

**Background:**

Biofilms, or colonies of uropathogen growing on the surface of indwelling medical devices, can inflict obstinate or recurring infection, thought-provoking antimicrobial therapy.

**Methods:**

This prospective analysis included 105 urine samples from catheterized patients receiving intensive care. Ensuing phenotypic identification, antibiotic sensitivity test was performed by modified Kirby–Bauer disc diffusion method following CLSI guidelines; MDR isolates were identified according to the combined guidelines of the European Centre for Disease Prevention and Control (ECDC) and the Centers for Disease Control and Prevention (CDC). Biofilm-forming uropathogens were detected by the tissue culture plate (TCA) method.

**Results:**

The predominant uropathogen in catheter-associated UTIs (CAUTIs) was *Escherichia coli* 57%, followed by *Klebsiella pneumonia* 15%, *Pseudomonas aeruginosa* 12%, *Staphylococcus aureus* 8%, *Enterobacter* spp. 3%, *Enterococcus faecalis*, *Acinetobacter* spp., and *Proteus mirabilis* 1.5%, of which 46% isolates were biofilm producers. Prime biofilm producers were *Escherichia coli* 33%, followed by *Klebsiella pneumoniae* 30%, *Pseudomonas aeruginosa* 20%, *Staphylococcus aureus* 10%, *Acinetobacter*, and *Enterobacter* 3.33%. Multidrug resistance associated with biofilm producers were greater than biofilm nonproducers. The Gram-negative biofilm producers found 96.15%, 80.76%, 73.07%, 53.84%, 53.84%, 46.15%, 19.23%, and 11.5% resistant to amoxyclave, ceftazidime, tetracycline, gentamicin, meropenem, nitrofurantoin, amikacin, imipenem, and fosfomycin, respectively. Gram-positive biofilm producers, however, were found 100% resistant to tetracycline, cloxacillin, and amoxyclave: 66.67% resistant to ampicillin while 33.33% resistant to gentamicin, ciprofloxacin, and nitrofurantoin.

**Conclusion:**

High antimicrobial resistance was observed in biofilm producers than non-biofilm producers. Of recommended antimicrobial therapies for CAUTIs, ampicillin and amoxicillin-clavulanate were the least active antibiotics, whereas piperacillin/tazobactam and imipenem were found as the most effectual for gram-negative biofilm producer. Likewise, amoxicillin-clavulanate and tetracycline were the least active antibiotics, whereas vancomycin, fosfomycin, piperacillin-tazobactam, and meropenem were found as the most effective antibiotic for Gram-positive biofilm producer. In the limelight, the activity fosfomycin was commendable against both Gram-positive and Gram-negative biofilm producers.

## 1. Background

Of nearly 40 percentile of all health care-associated infections, urinary tract infections (UTIs) are the foremost cause of infections; out of these, a bulky proportion, 80%, involve catheter-associated urinary tract infections (CAUTIs) [1]. The urinary catheters are routinely used in urology practice; albeit, advances in design and materials used, UTIs persist as the major snags, owing to the contamination of such indwelling devices [2]. Approximately, prior admission, 12 to 16% of adult hospital inpatients have an indwelling urinary catheter, however, known to be associated with high morbidity, high mortality, increased the length of hospital stay, and increased the cost of treatment [1–3]. Furthermore, the catheter-associated biofilm producers, preceding drug resistivity, and their thought-provoking infection control procedures have been reported in aforementioned studies, which raises our concern on CAUTIs and biofilm producers in our settings [4, 5].

Biofilms are the sessile polymicrobial communities attached to the substratum of biotic and abiotic surfaces and are sheathed within a self-produced extracellular polymeric matrix, that is, polysaccharides intercellular adhesin [2, 5, 6]. The extracellular matrix facilitates communications among the cells through biochemical signals—acyl-homoserine lactone in Gram-negative bacteria and oligopeptides in Gram-positive bacteria—in a phenomenon called as quorum sensing [7]. Not only the matrix precludes the pathogen against host defense but also attributes antimicrobial resistance, by subordinating antibiotic penetration, horizontal transmission of plasmid-associated drug-resistant gene, and altered microenvironment [6, 7]. In this standpoint, early detection of biofilm producers is crucial, to reduce the irrational antimicrobial burden proceeding antimicrobial resistance in the patient; hence, it would be an auxiliary in controlling device-associated infections in medical centers.”

The rationale of our study was to explicate bacterial etiologies, illuminate biofilm-associated resistivity patterns, and to endorse suitable antimicrobial therapy against biofilm producers in CAUTIs.

## 2. Material and Methods

### 2.1. Study Design and Setup

The cross-sectional study was conducted at the Department of Microbiology, Janamaitri Foundation Institute of Health Science (JFIHS), Nepal, over a period of six months. The study hospital is a referral centre with medical, surgical, gynecological, pediatric, geriatric, and other specialties.

### 2.2. Inclusion and Exclusion Criteria

The urine sample of all catheterized patients irrespective of gender and age between 12 and 70 years who met the criteria of CAUTI were included in the study. Nevertheless, noncatheterized patients, either nursed in ward or formerly under antimicrobial therapy at least 48 h prior catheter insertion and no more than two types of organism grown from the clinical sample, were considered as contaminated and consequently, excluded from the study.

### 2.3. Laboratory Methods

CAUTI was defined using a combination of clinical signs and symptoms and laboratory criteria as described by Stamm [2]. A total of 105 urine sample from the catheterized patient, admitted in intensive care units, were processed semiquantitatively by inoculating 0.001 ml of the specimen (by using a calibrated wire loop) onto the Cystine Lactose Electrolyte Deficient (CLED) agar for the isolation and identification of significant uropathogens [8]. Following the inoculation, the plates were incubated for 24 hours at 37°C in an aerobic atmosphere. The growth of a single organism with a count of ≥10^2^ colony-forming units (CFU)/ml was considered to represent as CAUTIs and was identified using appropriate routine identification methods including colony morphology, Gram stain, and an in-house set of biochemical tests [8].

### 2.4. Antimicrobial Susceptibility Testing

The susceptibility of bacterial isolates against recommended antibiotics was tested by the modified Kirby–Bauer disk diffusion method on Mueller Hinton agar (HiMedia, India) following guidelines of Clinical and Laboratory Standards Institute (CLSI), Wayne, USA [9]. Antibiotics that were tested in our study include amoxycillin clavulanate (amc 20/10 *μ*g), ampicillin (amp 10 *μ*g), amikacin (ak 30 *μ*g), ceftazidime (caz 30 *μ*g), ceftazolin (30 *μ*g), cefuroxime (cfm 30 *μ*g), ciprofloxacin (cip 5 *μ*g), cloxacillin (cox 30 *μ*g), cotrimoxazole (cot 25 *μ*g), fosfomycin (fo 200 *μ*g), gentamicin (gen 10 *μ*g), imipenem (imp 10 *μ*g), meropenem (mrp 10 *μ*g), nitrofurantion (300 *μ*g), piperacillin-tazobactam (pit 100/10 *μ*g), teteracycline (te 30 *μ*g), and vancomycin (VAN 30 *μ*g) (HiMedia Laboratories, India). Further, elucidations of antibiotic susceptibility results were made conferring to the zone size interpretative standards of CLSI [9]. MDR isolates, resistant to at least one antimicrobial from three different groups of first-line drugs, tested were identified according to the combined guidelines of the European Centre for Disease Prevention and Control (ECDC) and the Centers for Disease Control and Prevention (CDC) [10]. *Escherichia coli* ATCC 25922, *Staphylococcus aureus* (ATCC 25923), and *Pseusdomonas aeruginosa* (ATCC 27853) were used as a control organism for antibiotic susceptibility testing.

### 2.5. Detection of Biofilm Production

The detection of biofilm was done by tissue culture method/microtiter plate method (TCA), the gold standard method, as described as Christensen et al. [11]. In brief, the bacterial isolates from fresh agar plates were inoculated in 2 ml of BHI broth and incubated for 24 h at 37°C. The cultures were then diluted 1 : 40 with fresh medium (BHI broth supplemented with 1% glucose); 200 *µ*l of the sample was dispensed in the individual microtitration plate (AD Touch, apDianv) and incubated further 24 h at 37°C. With a gentle tapping, the content was removed further with a subsequent washing with phosphate buffer saline (pH 7.2) three times to remove free floating sessile bacteria. The adherent bacteria, biofilm producer, were fixed with sodium acetate (2%) and stained with crystal violet (0.1% w/v) for 10–15 min. The unbound crystal violet solution was removed with a triplicate washing with PBS, and the plate, then, was kept for drying. Finally, all wells were filled with 200 *µ*l ethanol (95%) to release dye from the well and Optical Density (OD) was taken at the wavelength of 630 nm. For a precision, the experiment was performed in triplicate two times. Average OD values of each test strain and negative control were calculated, and OD cut-off values (ODc) were assessed as described by Stepanovie et al. [12].

### 2.6. Data Analysis

The information regarding patients' profile and the results were entered into a computer program. Data analysis was carried out using the Statistical Package for Social Sciences (SPSS™) version 20.0 (IBM, Armonk, NY, USA) and presented in percentage base distribution.

### 2.7. Ethical Consideration

Written approval was taken from the Institutional Review Committee (IRC) of Janamaitri Foundation Institute of Health Science (JFIHS) after submitting and presenting the research proposal. Written informed consent was taken from every patient or their guardians before enrollment in the study.

## 3. Results

### 3.1. Patient Demographics

During the study period, a total of 105 urine specimens from the patients suspected with catheter-associated UTIs were processed. Among total clinical specimens, 61.9% (65/105) were found with a growth of at least one significant pathogen confirming the urinary tract infection (UTI). Female (43, 66.2%) was the most affected group, with predominant etiologies as *Escherichia coli* 56.9% (37/65) followed by *Klebsiella pneumoniae* 10 (15%), *Pseudomonas aeroginosa* 8 (12%), *Staphylococcus aureus* 5 (8%), *Enterobacter* spp 2 (3%), and *Enterococcus faecalis*, *Acinetobacter* spp., and *Proteus mirabilis* 1 (1.5% each) (Table 1).

### 3.2. Detection of Biofilm Producers

In the current study, 30 (46%) strains were *in vitro* positive for the biofilm production and 35 (54%) were negative for the biofilm production. Out of which (*n*=7, 9, and  14) were strong, moderate, and weak biofilm producer. *Escherichia coli* (33.33%) was found to be more biofilm producer followed by *Klebsiella pneumoniae* (30%), *Pseudomonas aeroginosa* (20%), *Staphylococcus aureus* (10%), and *Acinetobacter* spp. and *Enterobacter* spp. (3.33% each) (Table 2).

**Figure 1:**
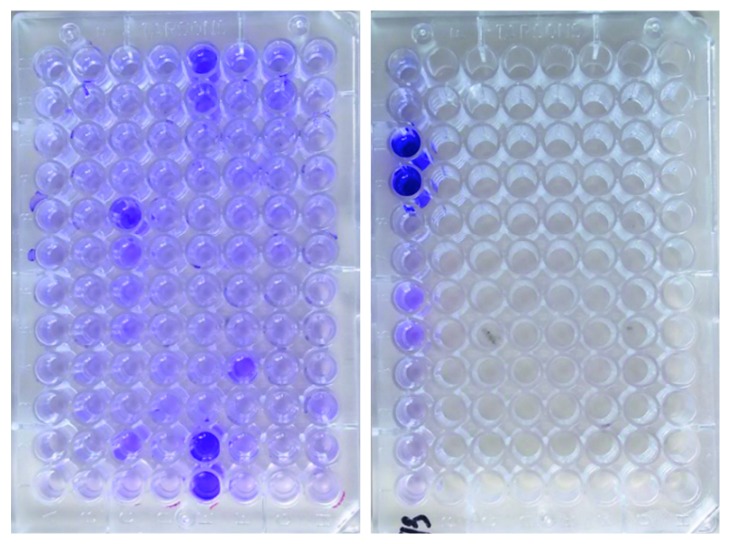
Microtitration plate showing the biofilm production by the TCA method.

Microtitration plate shows the biofilm production by TCA method as shown in Figure 1.

### 3.3. The Antimicrobial Resistant Pattern in Biofilm Producers and Nonproducers

Gram-negative biofilm producers, more than 90%, were resistant to ampicillin and amoxicillin-clavulanate, and nearly 94% of biofilm producer and nonproducer were found to be sensitive to fosfomycin. Besides, the greater percentile of antimicrobial resistivity was found to be associated with biofilm producers than biofilm nonproducers. The Gram-positive biofilm producers, 3 of 3 bacterial isolates, were resistance to amoxicillin-clavulanate, tetracyclin, and cloxacillin; nonetheless, the antibiotics—vancomycin, fosfomycin, meropenem, and piperacillin/tazobactam—were found to be effective even for the biofilm producers (Table 3).

The multidrug combination, in Gram-positive isolates and Gram-negative isolates, was found to be resistant to ciprofloxacin/cotrimoxazole/ampicillin (75%) and ampicillin/cloxacillin/teicoplanin (66.67%) (Table 4).

## 4. Discussion

Most aspects of the diagnosis, treatment, and prevention of CAUTI are influenced by the tenacity of biofilm-associated uropathogens [13]. Meanwhile, in patients with underlying diseases or under intensive care, the relevance of detection of biofilm producers is crucial since CAUTIs is a common nosocomial infection [1]. The prevalence of catheter-associated urinary tract infection was found to be 61.9%, with predominance in female sufferers (43, 66.2%). Similar prevalence was reported in a review of Nicolle from 15 developing countries like ours [14]. This might be due to the anatomical differences of urogenital organs—anal proximity and shorter urethra in female [15].

Our study revealed that the most common bacterial etiology contributing CAUTIs was *Escherichia coli* 57%, followed by *Klebsiella pneumoinae* 15%, *Pseudomonas aeruginosa* 12%, *S. aureus* 8%, *Enterobacter* spp. 3%, and *Enterococcus faecalis*, and *Acinetobacter* spp. and *Proteus mirabilis* (1.5% each) which was nearly similar to the findings Seif Elden Salwa et al. who found that *Escherichia coli* (50%) was predominantly acquired infection followed by *Klebsiella* spp. (30%), *Pseudomonas* spp. (8%), *Staphylococcus aureus* (8.6%), and *Proteus* spp. (4.6%) [16]. In a study, Niveditha et al. and Pramodhini reported 70% of *Escherichia coli* were isolated [17, 18]; in contrast, Ahmed Abdallah et al. reported 31% *Escherichia coli* in their study [19]. The recent prevalence study, however, depicted the predominance of the *Escherichia coli* attaining 79.1% leading UTIs [20]. Similarly, 46% of the bacterial isolates were detected as biofilm producers in the CAUTIs, ensuing with the TCA method. Similar findings were reported by Ghanwate et al. and Ahmed Abdallah et al. where the biofilm producers were 47.5% and 43.5%, respectively [19, 21]. Niveditha et al. and Reid et al., however, found the associated biofilm producers 60% and 73%, respectively, which matched with our study [17, 22].

In our study, 46% isolates were biofilm producers using the tissue culture plate method. Similar findings were observed by Safia Maqbool et al. and Ahmed Abdallah et al. where 47.5% and 43.5% uropathogenic isolates were biofilm producers, respectively; Niveditha et al. and Reid et al., however, found 60% and 73% biofilm producers as CAUTIs [17, 19, 22, 23]. The uropathogen, *Escherichia coli* 33%, emerges as a predominant biofilm producer followed by *Klebsiella* 30%, *Pseudomonas* spp. 20%, *S. aureus* 10%, and *Acinetobacter* and *Enterobacter* (3.33% each) in our settings. The findings were comparable with Sharma et al. 67.5% and Nivenditha et al. 60% for the predominant isolate—biofilm producing *Escherichia coli* [17, 24]. However, Ponnusamy et al. in their study revealed that of 100 *Escherichia coli* strains, 72 were biofilm-positive phenotype [25]. Conferring a degree of biofilm producers, in our study, 11%, 14%, and 21% were strong, moderate, and weak biofilm producers, whereas Mishra et al. disclosed, 1.5%, 10.5%, and 34.3% as strong, moderate, and weak biofilm producers in their study [4].

Antibiotic resistance patterns of biofilm producer and nonproducer were observed; sequentially, high resistance against tested antibiotics was attributed in biofilm producers. In Gram-negative bacterial isolates, the resistance against four groups of antibiotics, ampicillin, nitrofurantoin, tetracycline, and meropenem, was equated in biofilm producer versus non-biofilm producer isolates; the consecutive antibiotics were 92.3% versus 84.84%, 46.15% versus 15.15%, 73.07% versus 51.51%, and 53.84% versus 36.31%, respectively. Similarly, the antibiotics ampicillin, ciprofloxacin, nitrofurantion, and tetracycline were 66.67% versus 33.33%, 33.33% versus 0%, 33.33% versus 0%, and 100% versus 66.67%, respectively. Pramodhini et al., nonetheless, disclosed ampicillin (83.3% versus 60%), cephotaxime (73.3% versus 35%), norfloxacin (80% versus 60%), and nalidxic acid (93% versus 70%) from his study [18]. Furthermore, fosfomycin has shown promising in vitro activity against both Gram-positive and Gram-negative biofilm producers, as earlier experienced by Neuner et al., Mihailescu et al. and Marquès et al. in treating urinary tract infections [26–28].

Hence, the antimicrobial resistivity in the isolate possibly attributed with the biofilm productions. In Gram-positive isolates, the resistance against 3 groups of antibiotics amoxicillin-clavulanate, tetracyclin, and cloxacillin was equated in biofilm producer versus non-biofilm producer isolates; the consecutive antibiotics were 100% resistant in biofilm producers while 33.3%, 66.7%, and 66.77% were found to be resistant in non-biofilm producers. Similarly, MRSA, resistant pattern linked with the tenacity of biofilm producers, was observed by Ando et al. [29]. In biofilms, poor antibiotic penetration, nutrient limitation and slow growth, adaptive stress responses, and formation of persister cells are hypothesized to constitute a multilayered defense [30].

The backdrop of antimicrobial resistant in biofilm producers urges seeking of naive therapeutic alternatives despite conventional antibiotic therapies. In the meantime, to prevent or to combat these obstinate biofilm producers, small molecules (N-acetylcysteine, Ca2+ and Mg2+ chelators), and matrix-targeting enzymes (dispersin B, DNase I, proteinase K and trypsin), bactericidal and antiadhesion coatings (trimethylsilane, carboxybetaine methacrylate, organoselenium, heparin, and hyaluronic acid; polymer brush coatings; and furanones) were commenced successfully in developed nations [31]. Ironically, the indorsed therapeutic solutions are farther than reach in the developing countries like ours.

## 5. Conclusion

Biofilm producing bacteria are responsible for many recalcitrant infections and are notoriously hard to eradicate, owing to the possible acquisition of multidrug status. High antimicrobial resistance was observed in biofilm producing pathogens than nonproducers. Of recommended antimicrobial therapies for CAUTIs, ampicillin and amoxicillin-clavulanate were the least active antibiotics, whereas piperacillin/tazobactam, fosfomycin, and imipenem were found as the most effective antibiotics among Gram-negative biofilm producers. Likewise, among Gram-positive biofilm producer isolates; amoxicillin-clavulanate and tetracycline were the least active, whereas vancomycin, fosfomycin, piperacillin/tazobactam, and meropenem were found as the most effective antibiotic. In the limelight, the activity of fosfomycin was laudable against both Gram-positive and Gram-negative biofilm producers. Hence, an early identification of biofilm producers with subsequent detection of antibiotic resistivity pattern is mandatory, to improve the clinical management of CAUTIs when the patient requires an intensive care.

## Figures and Tables

**Table 1:** Etiological agent of catheter-associated UTIs.

Organism	Frequency	Percent
*Escherichia coli*	37	56.9
*Klebsiella pneumoniae*	10	15.4
*Pseudomonas aeroginosa*	8	12.3
*Staphyloccous aureus*	5	7.7
*Enterobacter* spp.	2	3.1
*Enterococcus faecalis*	1	1.5
*Proteus mirabilis*	1	1.5
*Acinetobacter* spp.	1	1.5
Total	65	100.0

**Table 2:** Degree of biofilm production by the bacterial isolates.

Organisms	Nonproducer (%)	Biofilm producer			Total biofilm producer (%)
		Strong producer	Moderate producer	Weak producer	
*Escherichia coli*	27 (77.14)	1	4	5	10 (33.33)
*Klebsiella pneumonia*	1 (2.86)	2	3	4	9 (30.00)
*Enterococcus faecalis*	1 (2.86)	0	0	0	0 (0.00)
*Pseudomonas aeroginosa*	2 (5.71)	3	1	2	6 (20.00)
*Proteus mirabilis*	1 (2.86)	0	0	0	0 (0.00)
*Staphylococcus aureus*	2 (5.71)	0	0	3	3 (10.00)
*Acinetobacter* spp.	0 (0.00)	1	0	0	1 (3.33)
*Enterobacter* spp.	1 (2.86)	0	1	0	1 (3.33)
Total	35 (100)	7	9	14	30 (100)

**Table 3:** Antibiotic susceptibility of biofilm producer and non-biofilm producers.

Antibiotics	Biofilm producer isolates (*n*=26)	%	Biofilm nonproducer isolates (*n*=33)	%	Resistance of all isolates (*n*=59)	%
*Gram-negatives isolates*						
Ampicillin	24	92.30	28	84.84	52	88.13
Ceftazolin	20	76.92	25	75.75	45	76.27
Gentamicin	14	53.84	11	30.30	25	42.37
Ciprofloxacin	19	73.07	23	69.69	42	71.18
Nitrofurantion	12	46.15	5	15.15	17	28.81
Amikacin	12	46.15	11	33.33	23	38.98
Amoxicillin-clavulanate	25	96.15	24	72.72	49	83.05
Piperacillin-tazobactam	7	26.92	7	21.22	14	23.72
Cefuroxime	20	76.92	24	72.72	44	74.57
Imipenem	5	19.23	1	3.03	6	10.16
Fosfomycin	3	11.5	1	3.03	4	6.77
Meropenem	14	53.84	12	36.36	26	44.06
Tetracyclin	19	73.07	17	51.51	36	61.01
Cotrimoxazol	17	65.38	21	63.63	38	64.40
Ceftazidime	21	80.76	21	63.63	42	71.18

Antibiotics	Biofilm producer isolates (*n*=3)	%	Biofilm nonproducer isolates (*n*=3)	%	Resistance of all isolates (*n*=6)	%

*Gram-positive isolates*						
Ampicillin	2	66.67	1	33.33	3	50
Gentamicin	1	33.33	0	0.00	1	16.67
Ciprofloxacin	1	33.33	0	0.00	1	16.67
Nitrofurantion	1	33.33	0	0.00	1	16.67
Amoxicillin-clavulanate	3	100	1	33.33	4	66.67
Piperacillin-tazobactam	0	0.00	0	0.00	0	0.00
Meropenem	0	0.00	0	0.00	0	0.00
Tetracyclin	3	100	2	66.67	5	83.33
Cloxacillin	3	100	2	66.67	5	83.33
Vancomycin	0	0.00	0	0.00	0	0.00
Fosfomycin	0	0.00	0	0.00	0	0.00

**Table 4:** Multidrug resistance isolates in biofilm producer and non-biofilm producer and multidrug resistance in Gram-negative isolates.

Multidrug combination	Biofilm producer isolates (*n*=12)	%	Biofilm nonproducer isolates (*n*=22)	%
*Multidrug resistance in Gram-positive isolates*				
AK, CIP, COT, AMP	6	50	7	31.81
AK, CIP, COT	7	58	8	36.36
CIP, COT, AMP	9	75	13	59.00
COT, AMP, AK	7	58	8	36.36
AMP, AK, CIP	7	58	7	31.81

Multiple drug combination	Biofilm producer isolates (*n*=3)	%	Biofilm nonproducer isolates (*n*=3)	%

*Multidrug resistance in Gram-positive isolates*				
AMP, COX, TE, NIT	1	33.33	0	0.00
AMP, COX, TE	2	66.67	1	33.33
COX, TE, NIT	1	33.33	0	0.00
TE, NIT, AMP	1	33.33	0	0.00
NIT, AMP, COX	1	33.33	0	0.00

AK: amikacin; CIP: ciprofloxacin; COT: cotrimoxazole; AMP = ampicillin; COX = cloxacillin; TE = tetracyclin; NIT = nitrofurantoin.

## Data Availability

The data used to support the findings of this study are available from the corresponding author upon request.
